# Latent Heterogeneity in the Impact of Financial Coaching on Delay Discounting among Low-Income Smokers: A Secondary Analysis of a Randomized Controlled Trial

**DOI:** 10.3390/ijerph19052736

**Published:** 2022-02-26

**Authors:** Erin S. Rogers, Elizabeth Vargas, Christina N. Wysota, Scott E. Sherman

**Affiliations:** 1 Department of Population Health, NYU Grossman School of Medicine, New York, NY 10016, USA; elizabeth.vargas@nyulangone.org (E.V.); christina.wysota@nyulangone.org (C.N.W.); scott.sherman@nyulangone.org (S.E.S.); 2 Department of Prevention and Community Health, Milken Institute School of Public Health, George Washington University, Washington, DC 20052, USA; 3 VA NY Harbor Healthcare System, New York, NY 10010, USA

**Keywords:** smoking cessation, delay discounting, socioeconomic health disparities

## Abstract

Low-income adults are significantly more likely to smoke, and face more difficulty in quitting, than people with high income. High rates of delay discounting (DD) may be an important factor contributing to the high rates of tobacco use among low-income adults. Future-oriented financial coaching may offer a novel approach in the treatment of smoking cessation among low-income adults. This secondary analysis (N = 251) of data from a randomized controlled trial examined the integration of future-oriented financial coaching into smoking cessation treatment for low-income smokers. Linear regression and finite mixture models (FMM) estimated the overall and the latent heterogeneity of the impact of the intervention versus usual care control on DD rates 6 months after randomization. Though standard linear regression found no overall difference in DD between intervention and control (β = −0.23, *p* = 0.338), the FMM identified two latent subgroups with different responses to the intervention. Subgroup 1 (79% of the sample) showed no difference in DD between intervention and control (β = 0.25, *p* = 0.08). Subgroup 2 (21% of the sample) showed significantly lower DD (β = −2.06, *p* = 0.003) among intervention group participants versus control at 6 months. Participants were more likely to be a member of subgroup 2 if they had lower baseline DD rates, were living at or below 100% of federal poverty, or were married/living with a partner. This study identified a group of low-income adults seeking to quit smoking who responded to financial coaching with decreased DD rates. These results can be used to inform future targeting of the intervention to individuals who may benefit most, as well as inform future treatment adaptations to support the subgroup of low-income smokers, who did not benefit.

## 1. Introduction

People living with low income (i.e., below 150–200% of the federal poverty level, FPL [1,2]) are significantly more likely to smoke than people with high income [3,4,5]. People with low income are as interested in quitting, but are less likely to be successful, than people with high income [3]. The income disparity in tobacco use has persisted over the past 50 years—pointing to a need for novel interventions that address the unique needs and barriers to quitting for low-income smokers [6,7,8].

Behavioral economics suggest that higher rates of delay discounting (DD) may be an important factor contributing to the increased initiation of tobacco use and difficulties quitting among low-income adults. DD is a phenomenon in which people prefer small, immediate rewards compared to larger, delayed rewards—meaning that they “discount” the value of future rewards [9]. Because of the phenomenon of DD, the consequences of unhealthy behaviors become increasingly less effective at influencing behavioral decision-making when they are delayed [9]. Bickel et al. have conceptualized DD as a “trans-disease process” with a neurobehavioral basis that contributes to a wide range of addictions (e.g., alcohol, stimulants, opioids) and other maladaptive behaviors, including problem gambling and overeating [10]. In the case of tobacco use, there is evidence that high DD rates during adolescence predispose people to initiate tobacco use [11]. Current adult smokers also consistently demonstrate higher DD rates than non-smokers and ex-smokers [12,13], and higher rates of DD are associated with lower intentions to quit, higher levels of nicotine addiction, and higher prospective risk of smoking relapse [14,15,16]. Moreover, DD is sensitive to addictive states, such that periods of nicotine deprivation can increase one’s preference for immediate cigarettes [17,18] and immediate monetary rewards [18]. Due to the consistent and reciprocal relationships between high DD rates and tobacco use, DD is a promising therapeutic target in the development of new smoking cessation interventions.

DD is also higher among people experiencing resource scarcity, such as low income [19]. The relationship between DD and income is not well understood, and is potentially bidirectional in nature. On the one hand, higher DD may be a pre-existing impulsivity trait that leads to money mismanagement and lack of engagement in long-term planning to obtain and grow income [20,21]. In the reverse direction, economic deprivation may increase DD by causing people to focus on their immediate needs for survival, leaving them depleted of the emotional and cognitive resources to resist immediate gratification, and prioritize delayed goals [19,22]. Therefore, the higher DD rates observed among adults with low income may be adaptive and driven, in part, sociocontextually by their limited financial resources. This has implications for the development of smoking cessation interventions for low-income smokers that aim to reduce DD. Low-income smokers attending to urgent issues (e.g., paying rent) have limited time or emotional and cognitive bandwidth to attend to issues that benefit the future, such as quitting smoking [19]. Thus, interventions that address financial hardship as a contextual driver of high DD rates may be needed for this population.

Scholten et al. published a 2019 systematic review of interventions designed to reduce DD in adults [23]. They concluded that single-session experimental manipulations involving “future thinking” or “connectivity to future self” are some of the most effective approaches to reducing DD in the short-term. In particular, they found that 83% of experiments that required participants to vividly imagine positive future events (called episodic future thinking, EFT) found significant reductions in DD, including in samples of adult smokers [23,24,25,26,27,28,29,30]. EFT can be especially effective at reducing DD when the imagined future events are emotionally positive, plausible, and personally relevant [30]. Recent evidence further suggests that financial-goal-related EFT (i.e., vividly imagining a future financial goal) can be more impactful at reducing DD than general EFT [31]. Moreover, a combination of EFT and health-goal-setting may produce the largest impacts on DD, compared to EFT or health-goal-setting alone [32]. However, of importance to the design of tobacco use interventions for low-income smokers, Stein et al. reported that in a laboratory setting, EFT and simulated economic scarcity independently impacted DD in opposing directions [33]. They found that EFT decreased DD, whereas simulated scarcity increased DD, with no interaction between EFT and scarcity [33]. Therefore, an intervention that employs one-time EFT without addressing participants’ underlying financial hardship may not be effective for low-income populations. Indeed, Scholten et al. [23] recommended extending time-limited EFT manipulations into longer-term, more comprehensive interventions.

Future-oriented financial coaching interventions offer the potential to reduce both financial hardship and long-term DD. Theodos et al. conduced a randomized controlled trial (RCT) testing community-based financial coaching, and found that participants randomized to receive financial coaching reported progress toward attaining financial goals, and had 0.4–0.5-point reductions in financial stress and financial dissatisfaction three months post-intervention [34]. Observational and case study research has similarly found that financial coaching has positive impacts on financial goal-attainment, and increases in budgeting and savings [35]. Our team recently conducted an RCT that tested an intervention that integrated future-oriented financial coaching into smoking cessation treatment for low-income smokers [36]. That trial found reduced markers of financial hardship at follow-up among participants randomized to the intervention group. With respect to DD outcomes, DeHart et al. reported that college students randomized to receive a semester-long financial education course had significantly lower DD at follow-up compared to participants who received an abnormal psychology course [37]. Black and Rosen found that an intensive money-management-based substance use intervention led to lower DD over time among psychiatric patients with a history of cocaine and/or alcohol use [38].

There are currently no published studies testing the impact of financial coaching on DD among low-income smokers. To address this empirical gap, we conducted a secondary analysis of our prior financial coaching RCT with the following aims and hypotheses:

Aim 1: To estimate the relationship between participants’ financial hardship, tobacco use, and DD at baseline.

**Hypothesis** **1** **(H1).** *Participants experiencing more severe financial hardship, lower motivation to quit, and greater number of cigarettes smoked per day would have higher DD rates at baseline.*

Aim 2: To estimate the impact of future-oriented financial coaching on participants’ DD rates at 6-months.

**Hypothesis** **2** **(H2).** *Participants randomized to the intervention would have reduced DD at follow-up compared to participants randomized to a control group.*

Prior research has found heterogeneous impacts of interventions on DD outcomes based on participants’ baseline characateristics [39]. Therefore, we used a finite mixture model (FMM) [40,41,42,43,44,45,46] approach to achieve the current analysis’ final aim:

Aim 3: To estimate and characterize latent heterogeneity in the impact of the financial coaching intervention on 6-month DD.

**Hypothesis** **3** **(H3).** *The impact of the intervention on DD at follow-up will vary based on participants’ baseline characteristics.*

## 2. Materials and Methods

### 2.1. Settings and Participants

The full methods and primary results of the parent trial are published elsewhere [28]. A total of 410 participants were recruited from two safety-net medical centers, and from the community, in New York City (NYC). People were eligible for participation if they lived in NYC (to be eligible to receive financial empowerment services from NYC); were aged >17 years; smoked a cigarette in the past 30 days; had an annual household income below 200% of the federal poverty level (FPL); spoke English or Spanish; and managed their own funds. People were excluded for pregnancy or breastfeeding. Following a baseline assessment, a research assistant randomized participants 1:1 to Intervention or Control groups. The current analysis focused on participants with complete data on all variables of current interest at baseline and follow-up (*n* = 251).

### 2.2. Treatment Conditions

#### 2.2.1. Intervention Group

Intervention participants received an evidence-based multi-session smoking cessation coaching program designed to help them develop an individualized quit plan [47,48,49,50,51]. The smoking cessation coaching included motivational and efficacy enhancement, identifying and overcoming smoking triggers, and addressing environmental barriers to quitting. Participants were also eligible to receive a free 4-week supply of nicotine replacement therapy (NRT; patch, gum, or lozenge) [52]. The Intervention integrated two future-oriented financial coaching components into the cessation coaching:

(1) Screening and Referral for Benefits and Financial Empowerment Programs: To improve underlying participants’ financial health, and reduce financial hardship, Intervention counselors screened participants for benefits programs in several domains, including child care, education, food, health care, housing, and legal aid. The counselors also offered to schedule participants an appointment with an NYC Financial Empowerment Center (FEC) to receive one-on-one or family coaching to help with major financial issues, such as financial literacy and efficacy, debt/credit relief, obtaining a bank account, emergency cash assistance, long-term planning, and completing taxes. FEC counseling is free and confidential for NYC residents, regardless of income or immigration status.
(2) Future-Oriented Money Management Coaching: Participants were also offered money management coaching that followed the best practices in financial coaching by working with participants longitudinally to develop and work toward client-centered future goals [35,53]. The coaching had two primary objectives: (1) to help participants create and maintain a household budget to meet short- and long-term future goals; and (2) to highlight and reinforce the link between tobacco cessation and the participant’s goals through the release of discretionary income spent on tobacco. The financial-goal-setting followed EFT principles by helping participants identify and imagine future goals that were emotionally positive, plausible, and personally relevant [23]. Participants were encouraged to set at least one short-term goal that could serve as an immediate reward for quitting. Tobacco spending and savings were discussed during each session to reinforce the link between quitting smoking and achieving one’s goals.

#### 2.2.2. Waitlisted Control Group

Participants randomized to the Control group received the intervention after a 6-month waiting period. During the waiting period, Control group participants could receive usual care smoking cessation services from their providers or from the community.

### 2.3. Data Collection and Measures

Participants completed an in-person baseline survey after enrollment (before randomization), and a follow-up survey by phone or in-person six months after randomization.

#### 2.3.1. Dependent Variable

Delay Discounting: We assessed DD with the 27-item Monetary Choice Questionnaire (MCQ). Each question on the MSQ asks whether the participant prefers smaller amounts of money today over delayed larger amounts of money (e.g., “Would you prefer $54 today, or $55 in 117 days?”) [54]. Using a logistic regression approach [55], we calculated each participant’s discounting rate (k) for small, medium, and large rewards. We then calculated each participant’s overall k, and transformed it using the natural log function prior to analysis.

#### 2.3.2. Independent Variables

Sociodemographics: The survey asked about the participant’s age, sex, race, ethnicity, level of education, place of birth, marital status, and employment status.

Tobacco use: Questions drawn from the Population Assessment of Tobacco and Health adult questionnaire assessed whether the participant smoked daily or on some days, their motivation to quit using a 0–10 scale, and the number of cigarettes smoked per day on a typical day as an indicator of nicotine addiction [56].

Financial hardship: Tucker-Seeley and Thorpe propose a model of financial hardship that distinguishes between *material*, *behavioral*, and *psychosocial* components of financial hardship [57]. Each component may demonstrate unique relationships with one’s health and well-being. The *material* component refers to one’s actual financial resources. The *psychosocial* component refers to how one feels about his or her resources. The *behavioral* component refers to what one does with his or her limited resources, such as purposeful economizing, or reducing spending on essentials. In alignment with this model, we assessed three types of financial hardship. To assess *material* hardship, we used a combination of annual household income and the number of people in the household to classify each participant as living at/below or above (i.e., 101–200%) the federal poverty level (FPL). We further assessed *material* hardship with items from the InCharge Financial Distress/Financial Well-being Scale (IFDWS, [58]) asking about one’s level of confidence in being able to afford a $1000 emergency, frequency of living paycheck-to-paycheck, and frequency of being unable to afford leisure activities (1–10 scales; 1 = high confidence/low frequency, and 10 = low confidence/high frequency).

To assess *behavioral* financial hardship, we used a question adapted from the International Tobacco Control Four-Country Survey measuring 30-day smoking-induced deprivation [59]: “In the last 30 days, has there been a time when the money you spent on cigarettes resulted in not having enough money for any of these items: housing, food, household utilities, health care, transportation, and necessary clothing?” (Yes/No).

To assess *psychosocial* financial hardship, we used IFDWS [58] items capturing one’s level of stress about personal finances in general, level of financial stress today, and worry about meeting monthly living expenses (1–10 scales; 1 = low stress/worry, and 10 = high stress/worry). Lastly, we used a question from the Health and Retirement Study [60] to assess the amount of control that participants felt they had over their financial situation (0–10 scale; 0 = “no control at all”, and 10 = “very much control”).

### 2.4. Analysis

We first summarized baseline participant characteristics using means, standard deviations, and proportions. To achieve Study Aim 1, we used multivariable linear regression with backward elimination (*p*-value to remove >= 0.10) to estimate the relationships between the study’s independent variables and participants’ DD rates at baseline. To achieve Study Aim 2, we estimated the effect of the intervention on DD rates at 6-month follow-up two ways. First, we used a standard linear regression model to compare DD between groups at 6-months, controlling for baseline DD and covariates. We then employed a finite mixture model (FMM [40]) to account for potential unobserved population heterogeneity that may impact the effect of the intervention on 6-month DD rates. The FMM approach first identified the presence of latent subgroups (components), and then, assigned a posterior probability to each participant of membership in the latent subgroups. To determine the optimal number of components in the FMM, we initially estimated models with one to three components, and evaluated the model fit using the Bayesian Information Criteria (BIC), where smaller values were preferred. Based on the BIC, we moved forward with specifying the final mixture model with two components. Once the mixture model identified the two latent subgroups, we used logistic regression to estimate the relationship between participants’ baseline characteristics and their posterior probability of membership in the two subgroups. We checked for multicollinearity in our independent variables, and variance inflation factors were found to be below 3.0 for all variables in the model.

## 3. Results

### 3.1. Study Sample

Table 1 displays the baseline characteristics of the sample. Participants’ mean age was 54 (SD = 11) years, 59% were male, 37% were immigrants, 45% were Black or African American, 41% were Latinx ethnicity, 59% had a high school education or less, and 77% were not employed. Nearly all (95%) participants were daily smokers. Participants smoked, on average, 11 cigarettes per day (SD = 7), and they reported high motivation to quit (M = 8.2, SD = 6.3). Sixty nine percent of the sample was living at or below 100% of the FPL, and 45% reported recent smoking-induced deprivation. Participants reported high levels of material and psychosocial financial hardship on the IDFDS. Participants had a mean overall discounting rate of −4.0 (SD = 2.1) on the MCQ.

### 3.2. Relationship between Participant Characteristics and Delay Discounting at Baseline

Table 2 shows the statistically significant associations between participants’ baseline characteristics and their overall DD rate at baseline. Immigrants had significantly lower DD rates than US-born participants (β = −0.72, *p* = 0.009). Financial locus of control and level of stress about one’s personal finances were negatively associated with DD. The more internal control that participants felt over their financial situation, the lower their DD rate (β = −0.12, *p* = 0.007), and the more stress that people felt about their personal finances in general, the lower their DD rate (β = −0.52, *p* = 0.002). Two material hardship variables were positively associated with DD. People living at or below the FPL had significantly higher rates of DD than people living between 101–200% of FPL (β = 0.55, *p* = 0.049). Participants who reported greater frequency in being unable to afford leisure activities had higher DD rates (β = 0.13, *p* = 0.010). The remaining independent variables were not significantly associated with DD (*p* > 0.05)

### 3.3. Effect of the Intervention on Delay Discounting

Table 3 shows the results of the regression model and the FMM. The standard regression model found no significant group difference in DD at 6-months (β (SE) = −0.23 (0.24), *p* = 0.34). In contrast, the FMM identified two latent subgroups, comprising 79.1% and 20.9% of the sample, with differing response to the intervention. There was no group difference in DD among members of Subgroup 1 (β (SE) = 0.25 (0.14), *p* = 0.08). Among members of Subgroup 2, the Intervention was associated with significantly lower DD at follow-up (β (SE) = −2.06 (0.69), *p* = 0.003). Participants were more likely to be in Subgroup 2 if they had lower baseline DD rates (aOR = 0.75, 95% CI 0.65–0.88), were married or living with a partner (aOR = 2.37, 95% CI 1.08–5.20), or were living at/below FPL (versus 101–200% FPL; aOR = 2.71, 95% CI 1.15–6.42).

## 4. Discussion

This study has several important findings. First, the baseline analysis revealed that the severity of participants’ material and psychosocial financial hardships were the primary correlates of DD in this sample of low-income smokers. This finding conflicts with perspectives that low-income smokers discount more steeply due to trait impulsivity [20,21], and supports the future investigation of sociocontextual determinants of DD in low-income populations. Much like nicotine deprivation can increase one’s preference for immediate cigarettes [17,18], modifiable economic deprivation may be a key driver of the high rates of monetary DD observed in low-income populations. In particular, participants in the current study with more income, and those who felt that they had more control over their financial situation, had lower DD rates (consistent with Plunkett and Buehner [61]). Interventions targeting DD as a therapeutic tool in low-income smokers should acknowledge and address participants’ financial hardship and perceptions of control.

Second, this study found that an intervention that integrated future-oriented financial coaching into smoking cessation treatment can reduce DD rates in low-income smokers. Given the strong relationships between DD and other unhealthy conditions [10] that are more likely to occur in low-socioeconomic groups (e.g., substance abuse, obesity), financial coaching has potential therapeutic value for reducing DD as a contributing factor for a range of health problems. The study further found that there were differential impacts of the intervention on DD based on participants’ baseline characteristics. These results can be used to guide future intervention targeting and adaptations. Participants with very low pre-treatment income, lower pre-treatment DD rates, or who were married/partnered responded to the intervention with reduced DD. Identifying the effective mechanisms of the intervention’s impact on DD in this subgroup requires future research. Even though the intervention included both financial coaching and smoking cessation treatment, it is unlikely that the smoking cessation treatment alone impacted DD. Athamneh et al. found EFT combined with health-related goal setting to be most impactful on DD [32]. Therefore, the integration of financial coaching into cessation treatment, and the reductions in financial distress experienced by intervention group participants [36], likely contributed to the reduced DD rates. Scholten et al. [23] summarized several additional pathways through which interventions can reduce DD that may have been present in the current intervention. These include participants perceiving an increased saliency, vividness, or personalization of future rewards; participants experiencing enhanced feelings of connectedness to their future self; or participants experiencing enhanced working memory.

This study has some limitations. The analysis is limited to participants who completed the parent RCT, which may limit generalizability to low-income smokers who are not interested in quitting, or to other settings. The study also focuses on just one aspect of smoking cessation (DD rates) among low-income smokers, and the reduced DD rates observed in the study may not translate to long-term changes in smoking behavior. Further, the two-group waitlist control design does not allow us to determine which intervention components impacted DD, or the mechanisms of these impacts. Despite these limitations, the study has many strengths, including its diverse sample, its novel intervention, its rigorous RCT design, and its use of validated measures.

## 5. Conclusions

Integrating future-oriented financial coaching into smoking cessation treatment was efficacious at reducing DD in a subgroup of participants characterized by lower pre-treatment DD, lower income, or a higher prevalence of being married/partnered. These results can be used to target the intervention to subgroups of low-income smokers most likely to benefit. Future research should seek to adapt the intervention to support subgroups who did not benefit from the intervention as it is currently designed. Future research is also needed to test whether reducing DD via financial coaching leads to increased abstinence rates among low-income smokers.

## Figures and Tables

**Table 1:** Baseline characteristics of study sample.

Variable	Total (*n* = 251)	Intervention (*n* = 118)	Control (*n* = 133)
	*n* (%) or Mean (SD)		
**Sociodemographics**			
Age	53.7 (10.8)	54.2 (10.8)	53.2 (10.8)
Immigrant	92 (36.7%)	43 (36.4%)	49 (36.8%)
Female	103 (41.0%)	46 (39.0%)	57 (42.9%)
Race			
Black/African American	112 (44.6%)	54 (45.8%)	58 (43.6%)
White	51 (20.3%)	24 (20.3%)	27 (20.3%)
American Indian/Alaskan Native	6 (2.4%)	1 (0.8%)	5 (3.8%)
Asian	5 (2.0%)	3 (2.5%)	2 (1.5%)
Other	91 (36.3%)	40 (33.9%)	51 (38.4%)
Latinx Ethnicity	102 (40.6%)	49 (41.5%)	53 (39.8%)
Highest level of education			
High school graduate/GED or lower	148 (59.0%)	71 (60.2%)	77 (57.9%)
Greater than high school/GED	103 (41.0%)	47 (39.8%)	56 (42.1%)
Marital status			
Married/living with partner	49 (19.5%)	20 (16.9%)	29 (21.8%)
Separated/divorced/widowed/never married	202 (80.5%)	98 (83.1%)	104 (78.2%)
Unemployed	193 (76.9%)	88 (74.6%)	105 (78.9%)
**Smoking characteristics**			
Smokes daily	238 (94.8%)	110 (93.2%)	128 (96.2%)
Cigarettes per day	11.3 (6.9)	10.6 (6.8)	12.0 (6.9)
Quit motivation (0–10 scale)	8.2 (6.3)	8.7 (8.6)	7.8 (2.7)
**Behavioral Financial Hardship**			
Smoking-induced deprivation	114 (45.4%)	55 (46.6%)	59 (44.4%)
**Material Financial Hardship**			
Living at or below 100% of FPL	174 (69.3%)	80 (67.8%)	94 (70.7%)
Frequency of getting by paycheck-to-paycheck (1–10 scale)	8.2 (2.6)	8.1 (2.8)	8.2 (2.5)
Confidence in affording $1000 emergency (1–10 scale)	3.8 (3.3)	3.7 (3.3)	3.8 (3.3)
Frequency of inability to afford leisure activities (1–10 scale)	6.6 (2.9)	6.4 (3.1)	6.7 (2.9)
**Psychosocial Financial Hardship**			
Stress about finances in general (1–10 scale)	6.4 (2.7)	6.3 (2.9)	6.4 (2.6)
Financial stress today (1–10 scale)	6.0 (2.9)	5.7 (2.9)	6.2 (2.8)
Worry about meeting monthly living expenses (1–10 scale)	6.2 (2.9)	6.1 (2.9)	6.3 (2.9)
Satisfied with present financial situation (1–10 scale)	3.9 (2.8)	4.2 (3.0)	3.7 (2.6)
Worry about current financial situation (1–10 scale)	6.8 (2.6)	6.7 (2.6)	6.9 (2.6)
Personal control over financial situation (1–10 scale)	6.5 (3.3)	6.1 (3.3)	6.9 (3.2)
**Delay Discounting—ln(k)**			
Overall	−4.0 (2.1)	−4.1 (2.0)	−4.0 (2.2)
Small	−3.8 (2.2)	−3.9 (2.0)	−3.8 (2.3)
Medium	−4.2 (2.3)	−4.2 (2.2)	−4.3 (2.4)
Large	−4.7 (2.2)	−4.8 (2.1)	−4.6 (2.3)

Note: GED = general education development. Delay discounting was measured with the 27-item Monetary Choice Questionnaire [54]. DD data presented are the natural log transformed discount rates (k).

**Table 2:** Significant associations between participant characteristics and delay discounting at baseline.

Variable	β (SE)	*p*-Value
Immigrant	−0.72 (0.27)	0.009
Personal financial locus of control	−0.12 (0.04)	0.007
Level of stress about personal finances in general	−0.17 (0.05)	0.002
Living at or below 100% FPL (versus 101–200% FPL)	0.55 (0.28)	0.049
Frequency of being unable to afford leisure activities	0.13 (0.05)	0.010

Note: FPL = federal poverty level. SE = standard error. Delay discounting was measured with the 27-item Monetary Choice Questionnaire [54]. Each participant’s discounting rate (k) was transformed with the natural log function (ln(k)) prior to analysis. Backward elimination excluded the following insignificant variables from the model: age, gender, race, ethnicity, cigarettes per day, motivation to quit, education, employment, marital status, smoking-induced deprivation, and the ramaining IFDWS items.

**Table 3:** Regression and finite mixture model (FMM) results estimating the impact of the intervention on delay discounting at 6-months.

	Standard Regression		FMM Subgroup 1		FMM Subgroup 2	
Outcome	β (SE)	*p*-Value	β (SE)	*p*-Value	β (SE)	*p*-Value
Intervention vs. Control	−0.23 (0.24)	0.34	0.25 (0.14)	0.08	−2.06 (0.69)	<0.01
Subgroup probability	--		79.1%		20.9%	

Note: Delay discounting was measured with the 27-item Monetary Choice Questionnaire [55]. Each participant’s discounting rate (k) was transformed with the natural log function (ln(k)) prior to analysis. SE = standard error. Models control for baseline ln(k) and covariates.

## Data Availability

De-identified data are available upon request from the corresponding author.

## References

[1] National Center for Children in Poverty United States Demographics of Low-Income Children. https://www.nccp.org/demographic/.

[2] United States Government: Community Services Block Grant Act ( 42 U.S. Code §9902(2)). https://uscode.house.gov/view.xhtml?path=/prelim@title42/chapter106&edition=prelim.

[3] Barbeau E.M., Krieger N., Soobader M.J. (2004). Working class matters: Socioeconomic disadvantage, race/ethnicity, gender, and smoking in NHIS 2000. Am. J. Public Health.

[4] Farrelly M.C., Nonnemaker J.M., Watson K.A. (2012). The consequences of high cigarette excise taxes for low-income smokers. PLoS ONE.

[5] Centers for Disease Control and Prevention (2011). Vital signs: Current cigarette smoking among adults aged >/=18 years—United States, 2005–2010. MMWR Morb. Mortal. Wkly. Rep..

[6] Bryant J., Bonevski B., Paul C., McElduff P., Attia J. (2011). A systematic review and meta-analysis of the effectiveness of behavioural smoking cessation interventions in selected disadvantaged groups. Addiction.

[7] Michie S., Jochelson K., Markham W.A., Bridle C. (2009). Low-income groups and behaviour change interventions: A review of intervention content, effectiveness and theoretical frameworks. J. Epidemiol. Community Health.

[8] Fu S.S., van Ryn M., Burgess D.J., Nelson D., Clothier B., Thomas J.L., Nyman J.A., Joseph A.M. (2014). Proactive tobacco treatment for low income smokers: Study protocol of a randomized controlled trial. BMC Public Health.

[9] Bickel W.K., Marsch L.A. (2001). Toward a behavioral economic understanding of drug dependence: Delay discounting processes. Addiction.

[10] Bickel W.K., Jarmolowicz D.P., Mueller E.T., Koffarnus M.N., Gatchalian K.M. (2012). Excessive discounting of delayed reinforcers as a trans-disease process contributing to addiction and other disease-related vulnerabilities: Emerging evidence. Pharmacol. Ther..

[11] Audrain-McGovern J., Rodriguez D., Epstein L.H., Cuevas J., Rodgers K., Wileyto E.P. (2009). Does delay discounting play an etiological role in smoking or is it a consequence of smoking?. Drug Alcohol Depend..

[12] Baker F., Johnson M.W., Bickel W.K. (2003). Delay discounting in current and never-before cigarette smokers: Similarities and differences across commodity, sign, and magnitude. J. Abnorm. Psychol..

[13] Sansone G., Fong G.T., Hall P.A., Guignard R., Beck F., Mons U., Pötschke-Langer M., Yong H.-H., Thompson M.E., Omar M. (2013). Time perspective as a predictor of smoking status: Findings from the International Tobacco Control (ITC) Surveys in Scotland, France, Germany, China, and Malaysia. BMC Public Health.

[14] Athamneh L.N., Stein J.S., Bickel W.K. (2017). Will delay discounting predict intention to quit smoking?. Exp. Clin. Psychopharmacol..

[15] Sheffer C.E., Prashad N., Lunden S., Malhotra R., O’Connor R.J. (2019). To smoke or not to smoke: Does delay discounting affect the proximal choice to smoke?. Subst. Use Misuse.

[16] Sheffer C., Mackillop J., McGeary J., Landes R., Carter L., Yi R., Jones B., Christensen D., Stitzer M., Jackson L. (2012). Delay discounting, locus of control, and cognitive impulsiveness independently predict tobacco dependence treatment outcomes in a highly dependent, lower socioeconomic group of smokers. Am. J. Addict..

[17] Mitchell S.H. (2004). Effects of short-term nicotine deprivation on decision-making: Delay, uncertainty and effort discounting. Nicotine Tob. Res..

[18] Field M., Santarcangelo M., Sumnall H., Goudie A., Cole J. (2006). Delay discounting and the behavioural economics of cigarette purchases in smokers: The effects of nicotine deprivation. Psychopharmacology.

[19] Mullainathan S., Shafir E. (2013). Scarcity: Why Having Too Little Means So Much.

[20] Hampton W.H., Asadi N., Olson I.R. (2018). Good Things for Those Who Wait: Predictive Modeling Highlights Importance of Delay Discounting for Income Attainment. Front. Psychol..

[21] Meier S., Sprenger C. (2010). Present-Biased Preferences and Credit Card Borrowing. AEJ Appl. Econ..

[22] Vohs K., Faber R. (2007). Spent Resources: Self-Regulatory Resource Availability Affects Impulse Buying. J. Consum. Res..

[23] Scholten H., Scheres A., de Water E., Graf U., Granic I., Luijten M. (2019). Behavioral trainings and manipulations to reduce delay discounting: A systematic review. Psychon. Bull. Rev..

[24] Radu P.T., Yi R., Bickel W.K., Gross J.J., McClure S.M. (2011). A mechanism for reducing delay discounting by altering temporal attention. J. Exp. Anal. Behav..

[25] Chivers L.L., Higgins S.T. (2012). Some observations from behavioral economics for consideration in promoting money management among those with substance use disorders. Am. J. Drug Alcohol Abuse.

[26] Atance C.M., O’Neill D.K. (2001). Episodic future thinking. Trends Cogn. Sci..

[27] Peters J., Büchel C. (2010). Episodic future thinking reduces reward delay discounting through an enhancement of prefrontal-mediotemporal interactions. Neuron.

[28] Daniel T.O., Said M., Stanton C.M., Epstein L.H. (2015). Episodic future thinking reduces delay discounting and energy intake in children. Eat Behav..

[29] Stein J.S., Wilson A.G., Koffarnus M.N., Daniel T.O., Epstein L.H., Bickel W.K. (2016). Unstuck in time: Episodic future thinking reduces delay discounting and cigarette smoking. Psychopharmacology.

[30] Olsen R. (2020). The Effect of Episodic Future Thinking on Delay Discounting. Ph.D. Thesis.

[31] O’Donnell S., Daniel T.O., Epstein L.H. (2017). Does goal relevant episodic future thinking amplify the effect on delay discounting?. Conscious. Cogn..

[32] Athamneh L.N., Stein M.D., Lin E.H., Stein J.S., Mellis A.M., Gatchalian K.M., Epstein L.H., Bickel W.K. (2021). Setting a goal could help you control: Comparing the effect of health goal versus general episodic future thinking on health behaviors among cigarette smokers and obese individuals. Exp. Clin. Psychopharmacol..

[33] Stein J.S., Craft W.H., Paluch R.A., Gatchalian K.M., Greenawald M.H., Quattrin T., Mastrandrea L.D., Epstein L.H., Bickel W.K. (2021). Bleak present, bright future: II. Combined effects of episodic future thinking and scarcity on delay discounting in adults at risk for type 2 diabetes. J. Behav. Med..

[34] Theodos B., Simms M., Treskon M., Stacy C., Brash R., Emam D., Daniels R., Collazos J. (2015). An Evaluation of the Impacts and Implementation Approaches of Financial Coaching Programs.

[35] University of Wisconsin-Madison Center for Financial Security (2015). Financial Coaching: Review of Existing Research.

[36] Rogers E.S., Rosen M.I., Elbel B., Wang B., Kyanko K., Vargas E., Wysota C., Sherman S.E. (2022). Integrating financial coaching and referrals into a smoking cessation program for low-income smokers: A randomized waitlist control trial. JGIM.

[37] DeHart W.B., Friedel J.E., Lown J.M., Odum A.L. (2016). The Effects of Financial Education on Impulsive Decision Making. PLoS ONE.

[38] Black A.C., Rosen M.I. (2011). A money management-based substance use treatment increases valuation of future rewards. Addict. Behav..

[39] Snider S.E., Deshpande H.U., Lisinski J.M., Koffarnus M.N., LaConte S.M., Bickel W.K. (2018). Working Memory Training Improves Alcohol Users’ Episodic Future Thinking: A Rate-Dependent Analysis. Biol. Psychiatry Cogn. Neurosci. Neuroimaging.

[40] Muthén B., Shedden K. (1999). Finite mixture modeling with mixture outcomes using the EM algorithm. Biometrics.

[41] Burgette L.F., Cabreros I., Han B., Paddock S.M. (2021). Appropriate analyses of bimodal substance use frequency outcomes: A mixture model approach. Am. J. Drug Alcohol Abuse.

[42] Rhew I.C., Kosterman R., Lee J.O. (2017). Neighborhood Typologies Associated with Alcohol Use among Adults in Their 30s: A Finite Mixture Modeling Approach. J. Urban Health.

[43] Hofmeyr A., Monterosso J., Dean A.C., Morales A.M., Bilder R.M., Sabb F.W., London E.D. (2017). Mixture models of delay discounting and smoking behavior. Am. J. Drug Alcohol Abuse.

[44] Azagba S., Wolfson M. (2018). E-cigarette use and quantity of cigarette smoking among adolescent cigarette smokers: A finite mixture model analysis. Drug Alcohol Depend..

[45] Zhang Z., Abarda A., Contractor A.A., Wang J., Dayton C.M. (2018). Exploring heterogeneity in clinical trials with latent class analysis. Ann. Transl. Med..

[46] Vest N.A., McPherson S., Burns G.L., Tragesser S. (2020). Parallel modeling of pain and depression in prediction of relapse during buprenorphine and naloxone treatment: A finite mixture model. Drug Alcohol Depend..

[47] Rogers E.S., Smelson D.A., Gillespie C.C., Elbel B., Poole S., Hagedorn H.J., Kalman D., Krebs P., Fang Y., Wang B. (2016). Telephone Smoking-Cessation Counseling for Smokers in Mental Health Clinics: A Patient-Randomized Controlled Trial. Am. J. Prev. Med..

[48] Rogers E.S., Fu S.S., Krebs P., Noorbaloochi S., Nugent S.M., Gravely A., Sherman S.E. (2018). Proactive Tobacco Treatment for Smokers Using Veterans Administration Mental Health Clinics. Am. J. Prev. Med..

[49] Sherman S.E., Link A.R., Rogers E.S., Krebs P., Ladapo J.A., Shelley D.R., Fang Y., Wang B., Grossman E. (2016). Smoking-Cessation Interventions for Urban Hospital Patients: A Randomized Comparative Effectiveness Trial. Am. J. Prev. Med..

[50] Young H.M., Lierman L., Powell-Cope G., Kasprzyk D., Benoliel J.Q. (1991). Operationalizing the theory of planned behavior. Res. Nurs. Health.

[51] Bandura A. (1998). Health promotion from the perspective of social cognitive theory. Psychol. Health.

[52] Tobacco Use and Dependence Guideline Panel (2008). Treating Tobacco Use and Dependence: 2008 Update.

[53] Collins J.M., O’Rourke C.M. (2012). The Application of Coaching Techniques to Financial Issues. J. Financ. Ther..

[54] Kirby K.N., Petry N.M., Bickel W.K. (1999). Heroin addicts have higher discount rates for delayed rewards than non-drug-using controls. J. Exp. Psychol. Gen..

[55] Wileyto E.P. (2004). Stata module to score the Monetary Choice Questionnaire using logistic regression. Stat. Softw. Compon..

[56] Hyland A., Ambrose B.K., Conway K.P., Borek N., Lambert E., Carusi C., Taylor K., Crosse S., Fong G.T., Cummings K.M. (2017). Design and methods of the Population Assessment of Tobacco and Health (PATH) Study. Tob. Control.

[57] Tucker-Seeley R.D., Thorpe R.J. (2019). Material–Psychosocial–Behavioral Aspects of Financial Hardship: A Conceptual Model for Cancer Prevention. Gerontologist.

[58] Prawitz A.D., Garman E.T., Sorhaindo B., O’Neill B., Kim J., Drentea P. (2006). Incharge Financial Distress/Financial Well-Being Scale: Development, Administration, and Score Interpretation. J. Financ. Couns. Plan..

[59] Siahpush M., Borland R., Yong H.H., Cummings K.M., Fong G.T. (2012). Tobacco expenditure, smoking-induced deprivation and financial stress: Results from the International Tobacco Control (ITC) Four-Country Survey. Drug Alcohol Rev..

[60] Michigan Survey Research Center (2018). Health and Retirement Survey—2018.

[61] Plunkett H.R., Buehner M.J. (2007). The relation of general and specific locus of control to intertemporal monetary choice. Personal. Individ. Differ..

